# Design, development and validation of a new laryngo-pharyngeal endoscopic esthesiometer and range-finder based on the assessment of air-pulse variability determinants

**DOI:** 10.1186/s12938-016-0166-1

**Published:** 2016-05-10

**Authors:** Luis F. Giraldo-Cadavid, Luis Mauricio Agudelo-Otalora, Javier Burguete, Mario Arbulu, William Daniel Moscoso, Fabio Martínez, Andrés Felipe Ortiz, Juan Diaz, Jaime A. Pantoja, Andrés Felipe Rueda-Arango, Secundino Fernández

**Affiliations:** School of Medicine, University of Navarra, Irunlarea 1, 31080 Pamplona, Navarra Spain; School of Medicine, University of La Sabana, Autonorte de Bogotá, Km 7, Campus Puente del Común, Chia, 250001 Cundinamarca Colombia; School of Engineering, University of La Sabana, Autonorte de Bogota, Km 7, Campus Puente del Común, Chia, 250001 Cundinamarca Colombia; School of Sciences, University of Navarra, Irunlarea 1, 31080 Pamplona, Navarra Spain; Statistical Consulting Office, University of Santo Tomas, Carrera 9 # 51-11, Bogota, Colombia; Departamento de Medicina Interna, School of Medicine, University of La Sabana, Autonorte de Bogota, Km 7, Campus Puente del Comun, Chia, 250001 Cundinamarca Colombia

**Keywords:** Accuracy, Airway, Biomedical engineering, Biomedical equipment, Biomedical imaging, Bronchoscopy, Calibration, Deglutition, Endoscopes, Esthesiometer, Fiber lasers, Larynx, Mechanoreceptor, Medical diagnosis, Optical fibers, Pharynx, Range finder, Reflex, Reliability, Repeatability and reproducibility of results, Sensory thresholds, Telemeter

## Abstract

**Background:**

Laryngo-pharyngeal mechano-sensitivity (LPMS) is involved in dysphagia, sleep apnea, stroke, irritable larynx syndrome and cough hypersensitivity syndrome among other disorders. These conditions are associated with a wide range of airway reflex abnormalities. However, the current device for exploring LPMS is limited because it assesses only the laryngeal adductor reflex during fiber-optic endoscopic evaluations of swallowing and requires a high degree of expertise to obtain reliable results, introducing intrinsic expert variability and subjectivity.

**Methods:**

We designed, developed and validated a new air-pulse laryngo-pharyngeal endoscopic esthesiometer with a built-in laser range-finder (LPEER) based on the evaluation and control of air-pulse variability determinants and on intrinsic observer variability and subjectivity determinants of the distance, angle and site of stimulus impact. The LPEER was designed to be capable of delivering precise and accurate stimuli with a wide range of intensities that can explore most laryngo-pharyngeal reflexes.

**Results:**

We initially explored the potential factors affecting the reliability of LPMS tests and included these factors in a multiple linear regression model. The following factors significantly affected the precision and accuracy of the test (P < 0.001): the tube conducting the air-pulses, the supply pressure of the system, the duration of the air-pulses, and the distance and angle between the end of the tube conducting the air-pulses and the site of impact. To control all of these factors, an LPEER consisting of an air-pulse generator and an endoscopic laser range-finder was designed and manufactured. We assessed the precision and accuracy of the LPEER’s stimulus and range-finder according to the coefficient of variation (*CV*) and by looking at the differences between the measured properties and the desired values, and we performed a pilot validation on ten human subjects. The air-pulses and range-finder exhibited good precision and accuracy (*CV* < 0.06), with differences between the desired and measured properties at <3 % and a range-finder measurement error of <1 mm. The tests in patients demonstrated obtainable and reproducible thresholds for the laryngeal adductor, cough and gag reflexes.

**Conclusions:**

The new LPEER was capable of delivering precise and accurate stimuli for exploring laryngo-pharyngeal reflexes.

## Background

The objective exploration of laryngo-pharyngeal tract mechano-sensitivity (LPMS) is essential for the study, diagnosis and management of diseases affecting the functionality of the upper aerodigestive tract. In fact, the central nervous system controls many of the varied functions of the upper aerodigestive tract using the information provided by the mechanoreceptors of this tract’s mucosa [1]. In the human pharynx, mechanoreceptors of the pharyngeal wall replace muscle spindles for proprioception [2], and these receptors provide crucial information regarding pharyngeal movements for swallowing, breathing, and voice production, as well as airway protection and patency [2, 3]. Sensory alterations of the laryngo-pharyngeal tract, whether by hyposensitive or hypersensitive states, have been implicated as major underlying pathophysiological mechanisms of highly prevalent human diseases and may have a great impact on mortality.

Obstructive sleep apnea (OSA) patients have lower sensory capacity (higher sensory thresholds) throughout the laryngo-pharyngeal tract [4, 5], which impairs the reflex response of the central nervous system to regulate the tone of the upper airway dilatator muscles to maintain airway patency [4]. OSA is a major risk factor for cardiovascular diseases, which are the leading cause of death throughout the world [6, 7]. In developed countries, the number of years of life lost (YLLs) due to premature mortality as a result of OSA is estimated to be approximately 41 million per year, which was estimated from a total of 256 million YLLs resulting from all-cause mortality [7] and the population attributable fraction (PAF) of OSA among all deaths calculated from the meta-analysis conducted by Wang (PAF = 0.16) [6].

Laryngo-pharyngeal sensory deficits are a major pathophysiological mechanism of oropharyngeal dysphagia [8–11] especially in patients with stroke [9, 12–14]. The prevalence of oropharyngeal dysphagia in the general population is approximately 8.4 % [15]. In dysphagia patients, sensory deficits are predictors of aspiration of food into the lower airways and lungs [9, 10, 13] and might predict pneumonia, particularly aspiration pneumonia [11, 13]. Pneumonia is the 6th largest cause of death throughout the world [7], with more than 6 million YLLs because of pneumonia in developed countries. Sensory evaluations may help better select patients for gastrostomy and prevent pneumonia in dysphagic stroke patients [16].

The sensitivity of the laryngo-pharyngeal tract is not just important in cases of decreased sensation, there is growing evidence that a substantial number of patients affected by hypersensitivity states, such as irritable larynx syndrome [17–19], cough hypersensitivity syndrome [17, 20, 21], and amyotrophic lateral sclerosis [22], have decreased laryngo-pharyngeal sensory thresholds for cough and gag reflexes.

Furthermore, there is promising preliminary evidence for the efficacy of interventions to improve sensory impairments of the upper aerodigestive tract, such as using certain flavors, molecules and electrical stimulation [23–26]. Additionally, certain rehabilitation maneuvers are able to modulate hyper-reactive reflexes [17]. These interventions are either currently under investigation or will be investigated in the near future to reverse the aforementioned conditions [4, 17, 23, 24]. All of these interventions would benefit from objective and comprehensive tests of LPMS to evaluate their effectivity.

These sensory alterations may be objectively measured by a sensory meter or esthesiometer [27, 28]. A laryngo-pharyngeal esthesiometer must be able to provide precise (low variability or low random error) and accurate (low systematic error) stimuli in the range of intensities required to activate the laryngopharyngeal mechano-receptors responsible for the varied reflexes of this tract.

Aviv has been a pioneer of the clinical exploration of LPMS via air-pulse stimuli as part of the Fiberoptic Endoscopic Evaluation of Swallowing [29–31]. Aviv developed a device and standard technique for determining the psychophysical sensitivity of the laryngo-pharyngeal tract [29, 30] and the laryngeal adductor reflex threshold (LART) [31]. This device consists of a circuit board, compressor, pressure regulator, solenoid valve, valve driver, pressure transducer and transducer circuit and display [32]. However, the high repeatability observed by Aviv [29, 31] has not been replicated among less expert observers [33]; in addition, the reproducibility has been poor, even among skilled observers [33], which has limited the clinical application of this test.

Hammer found that the commercial version of Aviv’s device produced an error between the desired and delivered pressure level at a rate of 20 % and failed to deliver a stimulus at a rate of 17 % [34]. Hammer developed a new air-pulse generator for exploring LPMS, and although it improved the reliability of the air-pulses generated by the previous commercial device, Hammer’s device has not been commercialized [34]. Hammer’s device consists of similar elements of Aviv’s device except for the compressor, which has been replaced by an air cylinder, and a circuit control box, which provides control over the timing, duration and intensity of the stimulus and permits a greater range of stimulus durations and intensities [34]. Hammer also developed a method of improving the reliability of endoscopic distance estimations based on visualizing the individual blood vessels within the vocal cords and occupying at least 50 % of the area of the monitor by the arytenoids [34]. However, Hammer has not developed the technological improvements required to control the target distance [34] and has not published (to date) any studies on his device validating its precision and accuracy or reporting on its random and systematic errors.

Thus, only Aviv and Hammer have developed devices aimed to explore LPMS, and only Aviv's device has been commercialized. Both devices have been designed to assess the laryngeal adductor reflex threshold and psychophysical sensitivity and are not appropriate for assessing reflexes that require greater stimulus intensity, such as the cough and gag reflexes. In addition to a lack of target distance control, other factors may potentially affect the precision and accuracy of the superficial pressure of the air-pulses over the laryngo-pharyngeal mucosa, although they have not been explored experimentally. These factors include a lack of standardization of the tubes conducting the air-pulses, changes in the distance or angle between the endoscope distal end and the point of impact over the mucosa, and clinical factors such as frequent laryngeal movements and the amount and thickness of secretions. All of these factors may have a strong influence on the inter- and intra-observer variability of the sensory measurements.

The above mentioned issues indicate that there are currently limitations in LPMS measurements, and a precise and accurate test for exploring LPMS in the study, diagnosis and therapy of the aforementioned conditions is needed. Therefore, we performed a study to design, develop and validate a new air-pulse laryngo-pharyngeal endoscopic esthesiometer and range-finder (LPEER) based on evaluations and the control of air-pulse variability determinants and on intrinsic observer variability and subjectivity determinants of the distance, angle and site of the stimulus impact. In the first step, we explored the potential factors affecting the reliability of the stimuli used to measure the LPMS, and we then designed and developed a LPEER consisting of an air-pulse generator and an endoscopic laser range-finder. The LPEER was designed to control all of these factors and obtain random and systematic errors in the stimulus pressures and durations lower than 10 % (the range-finder aimed to control observer variability to determine the site, distance and angle of stimulus impact). Additionally, the LPEER was designed for exploring the LART as well as laryngeal reflexes that require greater stimulus intensities, such as the cough reflex (CRT) and gag reflex threshold (GRT). Finally, we aimed to assess the precision and accuracy of the stimuli generated by the new device and perform a pilot validation of the LPEER in a group of human subjects evaluated by two examiners with different levels of expertise.

## Methods

### Determination of the factors affecting the superficial pressure of air-pulses

The superficial pressure exerted by the air-pulses on the mechano-receptors of the laryngo-pharyngeal tract is the principal stimulus characteristic responsible for the activation of these mechanoreceptors [35]; however, the duration of the air-pulse may also have an effect [34].

A new laryngo-pharyngeal esthesiometer must be able to control all of the external factors that affect the pressure acting on the mucosa. These factors can be grouped into three large categories:

1. Pulse generation. The device must be able to produce a precise and accurate pulse-generation cycle, with a high repeatability and control of the different parameters of the problem. In particular, the applied pressure and the duration of the air-pulse [34] are parameters that must be tightly controlled. Furthermore, the reliability of the device has to be very high, thus, the number of failures has to be negligible. In the ideal scenario, the generated pulses have to be identical. In the design of our system, our goal was to achieve a maximum variability of 10 % on any of these parameters.
2. Generated flow. The endoscope distal end and the mucosa cannot be in contact with each other as this will produce a permanent stimulus, however, they must be relatively close to avoid damping of the air-pulse before it reaches the wall. For any air flow that we could produce, there is a critical distance where the air-pulse produces its maximum effect. The objective is to select an air recirculation that produces the same pressure on the mucosa for a broad range of distances. This can be obtained for some families of flows that are extremely stable.
3. Geometrical factors: distance and incidence angle. Other factors that can affect the stimulus response are how and where the air-pulse will impact the wall. The device must provide the observer with a screen providing a marked place where the air-pulse will stimulate the mucosa, the observer can then verify that the pulses arrive as normal to the surface as possible. Finally, the system should provide a clear estimation of the distance from the distal end of the endoscope to the surface so that the observer can standardize the distance of stimulus delivery.

These factors were experimentally evaluated by examining their capacity to determine the superficial pressure and were carefully taken into account on the design of the LPEER. The LPEER consists of an air-pulse generator that ensures an accurate control of the parameters defined in groups 1 and 2. It also has an endoscopic laser range-finder to control the factors in group 3. In the following sections we will discuss how these factors were evaluated, controlled, and incorporated into the final LPEER version. The first step for performing such an evaluation is the design and manufacture of an air-pulse generator.

### Air-pulse generator design and manufacture

As discussed above, the flow that leaves the distal end of the endoscope has to remain stable for a long distance (typically a few millimeters) compared to the tube diameter. This flow can be stabilized if it transports some physical magnitude whose diffusion is scarce, for example the vorticity [36]. A flow candidate that appears naturally is a vortex ring [36], a structure that can propagate on free air for distances hundreds of times larger than its diameter. The general features of this flow have been largely studied, as well as its interaction with the walls [37] and the effect of the impact angle [38]. Although a classical vortex ring was not possible for various practical reasons (e.g., due to the small size of the tube, the transport of momentum would be very small), we produced a similar flow (Fig. 6) acting with a square wave on the output valve that opens the air flow for a short period of time (typically 0.1 s). We have verified that the produced flow is stable for a long distance, and we visualized this using a chemical fog.

**Fig. 1:**
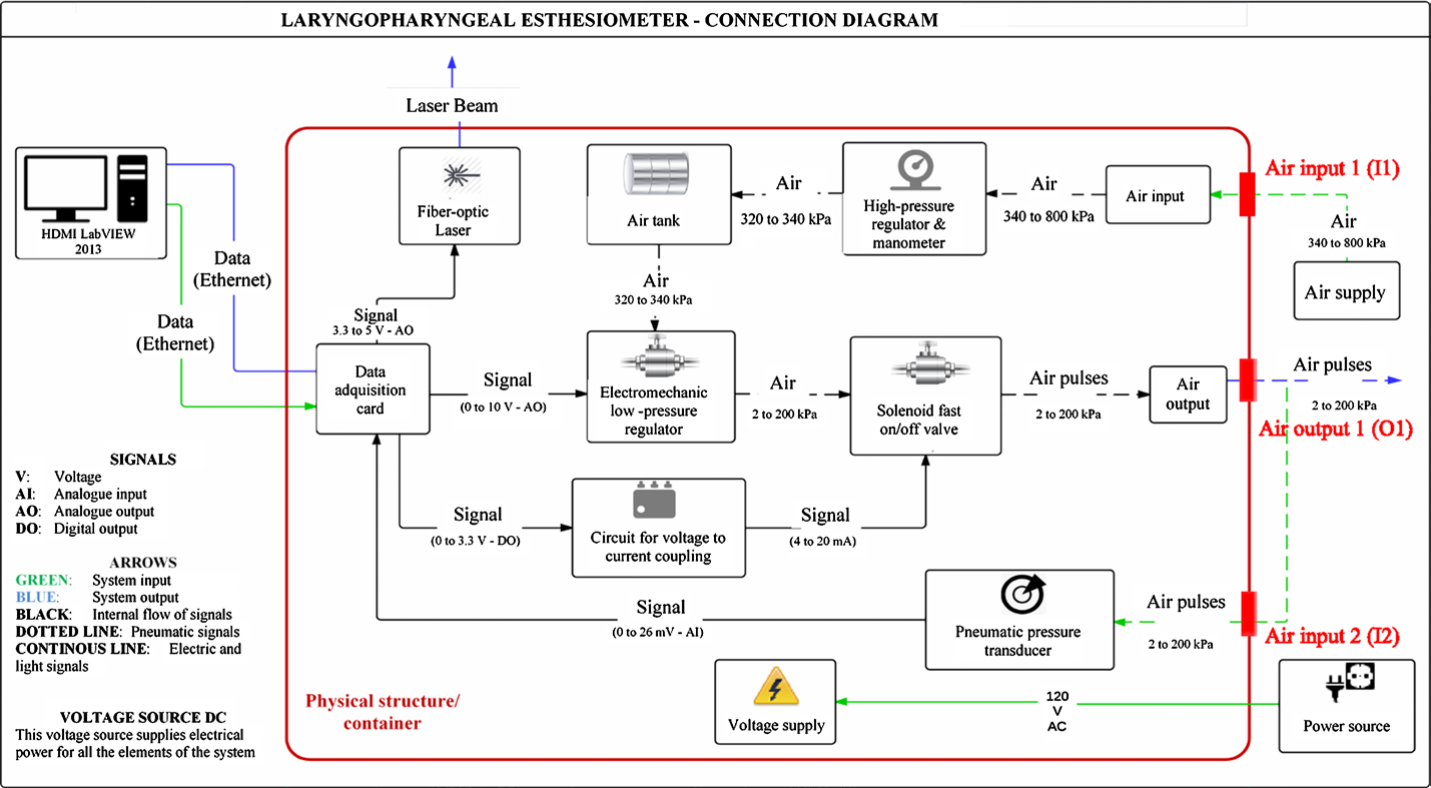
Air-pulse generator block diagram

**Fig. 2:**
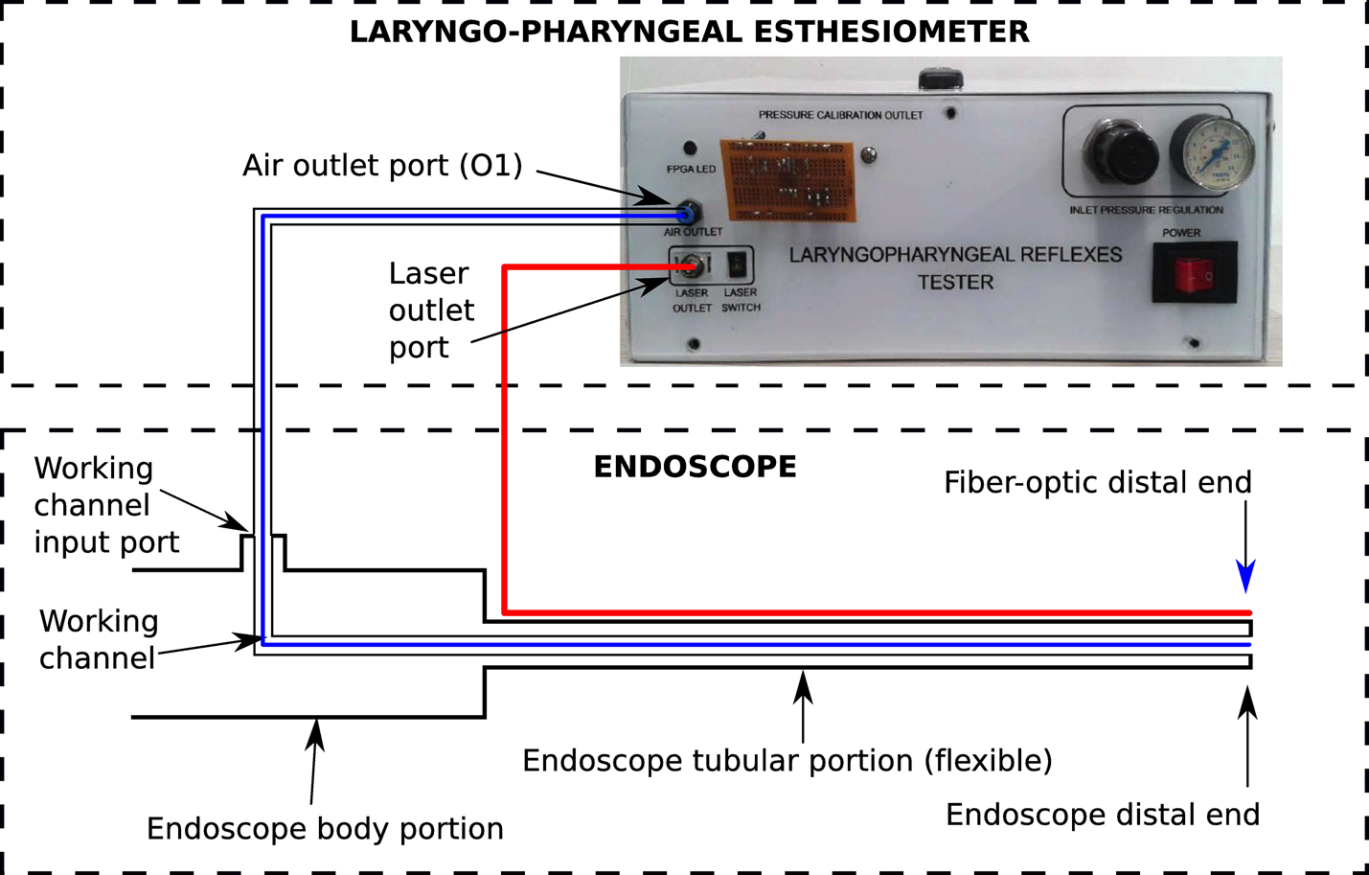
Diagram of the LPEER and endoscope assembly, where the air-pulse path (*blue*) and the optical fiber (*red*) represent a typical configuration

**Fig. 3:**
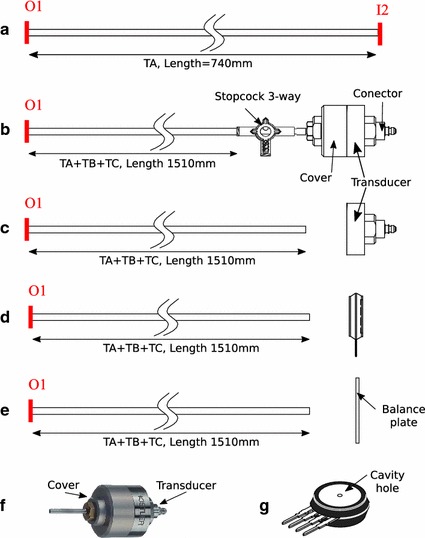
Sketch of the different configurations to measure pressures. **a** Calibration setup, where the tube is connected to the calibration port I2 (see Fig. 1). **b** Pressure drop measurement using the Kistler probe 7261 using a 3-way stopcock open to the atmosphere. **c** Assembly to measure the pressure of the air-pulses and the effect of the distance and impact angle. **d** Assembly similar to that used by Aviv and Hammer to measure the air-pulses [29, 31, 34]. **e** Assembly to measure the impact force (pressure) using a precision balance. In this last case, the balance plate is *horizontal*, and the air-pulses arrive from the *top*. **f** Picture of the Kistler sensor, where the cover and the transducer can be distinguished. **g** Sensor MPX2010D, where the air-pulse impacts the cavity hole that is placed in face of the distal end in the Aviv and Hammer studies configuration (**d**)

To achieve a good control over the parameters affecting the precision and accuracy of the pulses, one must govern the physical parameters that generate the flow. These physical parameters include the pressure difference applied and the resistance of the tube, the distal end of which is open to the atmosphere, and the proximal end is connected to the output port of the LPEER (labeled as O1 on Figs. 1, 2, 3). This resistance depends on the geometrical dimensions of the flexible tubes (diameter and length of the various sections) and the fluid (medical air).

The pressure drop through this tube can be described using the Darcy-Weisbach equation [39], which we used in the framework of a compressible fluid. Taking all these factors into account, the requirements for the pressure drop imply that the pressure at the outlet port should vary up to 200 kPa in order to attain pressures on the impact area in the range of 10 mmHg, or a total force of 10 mN.

The air-pulse generator has the following design specifications:

1. Generation of air-pulses ranging from 2 to 200 kPa to be delivered to the input port of the bronchoscope working channel (labeled as O1 on Figs. 1, 2, 3). This pressure range at the input port of the bronchoscope can produce air-pulses with intensities equivalent to 1–100 mmHg in the measurement methods of Aviv and Hammer (as will be discussed below); these air-pulses can obtain the LART as well as reflex thresholds that require greater stimulus intensities [29, 31, 34].
2. Period of 3000 ms to avoid interference with neuron refractory periods and permit the pressure inside the pneumatic system to stabilize at atmospheric pressure between pulses (to avoid summation of pressures of adjacent air-pulses).
3. Duration of air-pulses ranging from 50 to 1000 ms to obtain the LART as well as reflex thresholds that require greater stimulus durations [28, 29, 31, 34].

The air-pulse generator (Fig. 1) is an electromechanical device that receives air provided by an external medical air supply via an inlet (I1) and is regulated by an analog high-pressure regulator and manometer. This pressure regulator decreases the supply pressure to the pressure required by the electromechanical low-pressure regulator (300–400 kPa); the air then flows into a buffer air tank that supplies air downstream and is regulated by the electromechanical pressure regulator, which regulates the air pressure to 2–200 kPa. The air then flows into a fast on/off solenoid valve, which regulates the duration and period of the air-pulses. The opening of the electromechanical pressure regulator is controlled by voltage, modifying the potential difference applied to the valve from 0 to 10 V. Regulated air-pulses of a predetermined pressure, duration, and period are then transmitted through the port (O1): this output can be connected to the calibrating port (I2) through a tube (T_A_) with known characteristics (length: 740 mm, internal diameter: 2.6 mm) or to the working channel of the endoscope. The tubing in this case has three parts: the tube cited above (T_A_) connects the apparatus to an adaptor (T_B_) with a length of 173 mm and an internal diameter of 1.5 mm, which connects the line to the working channel of the endoscope T_C_ (length: 600 mm, diameter: 1.2 mm). This arrangement allows for the delivery of the pulses to the patient subjected to the sensory test (Fig. 1).

To reach the required pressures in O1 mentioned above (from 2 to 200 kPa), the electromechanical regulator requires a supply pressure of at least 300 kPa. This supply pressure may be provided by hospital air supply, oxygen cylinders (either small or large cylinders) or an external compressor (the compressor having the disadvantage of noise generation and associated patient discomfort because most small and silent compressors does not reach pressures above 200 kPa). We considered it impractical to incorporate the compressor to the LPEER because most tests are going to berformed in places with pipeline oxygen or oxygen cylinder availability, which do not generate noise.

The electrical connections are centralized in a controller that is wired and programmed to acquire data and control the functions of various components to control the operation of the electromechanical pressure regulator (i.e., its operating voltage) and the solenoid valve. The controller is also in operative communication with a computer via wired connections (Ethernet links), which are used to send and receive data or control commands from the computer via a user-machine interface. A software program based on LabVIEW 2013 (National Instruments Corporation, Austin, TX, USA) and responsible for reading and writing the digital and analog outputs and inputs is embedded in the controller (Fig. 1). The assembly of the LPEER and the endoscope is depicted in Fig. 2.

### Experimental assembly for measuring air-pulse characteristics

We used five different setups to determine the air-pulse pressure in various configurations. In Fig. 3 we present a sketch of each of these arrangements.

The first setup (Fig. 3a) corresponds to the configuration used to calibrate the pressure at the output of the electric valve. The output port (O1) and the input port (I2) are connected through tube T_A_. The port I2 is connected to a pressure sensor that allows the calibration and correct operating conditions of the electric valve.

The second arrangement (Fig. 3b) consisted of the connection of tubes T_A_ + T_B_ + T_C_ conducting the air-pulses from the air-pulse generator to an ultrahigh sensitive low-pressure transducer (Kistler, 7261, with a charge amplifier type 5015; Kistler Group, Winterthur, Switzerland) through a 3-way stopcock (Baxter International Inc., Deerfield, IL, USA), leaving all 3 ways open (Fig. 3b) to measure the outlet pressure and pressure drop. The desired pressures corresponded to those required to explore the LART [31], which is a series of air-pulses ranging from 0.13 to 1.33 kPa (1–10 mmHg). The duration of the valve opening for each pulse was set constant at 50, 100, 135, and 150 ms [29, 31, 34].

The third arrangement (Fig. 3c) was used to determine the characteristics of the pulses (repeatability, pulse duration, and pulse shape) with the same Kistler transducer, but uncovered. The superficial pressure on the surface of impact was also measured at different distances (1–10 mm) and angles (0°–60°). The air-pulse dimensions were measured by examining the change in the superficial pressure when interposing diaphragms with decreasing diameters (from 25 to 1 mm) were placed between the tube and the sensor.

The fourth setup (Fig. 3d) was used to determine the pressure in a manner similar to Aviv and Hammer’s LART experiments [29, 31, 34]. We utilized a MPX2010D Pressure Sensor (FREESCALE Semiconductor, Austin, TX, USA). The MPX2010D was placed and aligned at a 2-mm distance from the exit of the tube conducting the air-pulses, with the help of a M-460P-XYZ Peg-Joining Linear Stage (Newport, Irvine, CA, USA) and a high precision micrometer (Ball stage 45MM-X-CNTR-SOL, Edmund Optics Inc., Barrington, NJ, USA). We used an MPXV5004 sensor (FREESCALE Semiconductor, Austin, TX, USA) to measure the air-pulses needed for the CRT and GRT because these reflexes require stimuli above the range of the measurement of the MPX2010D.

Finally, a fifth arrangement (Fig. 3e) was used where the air-pulses impacted in the normal direction to the plate of a precision balance with a 100 mm pan diameter (Precisa BJ 100 M, Precisa Gravimetrics AG, Dietikon, Switzerland). This allowed us to characterize the air-pulses in terms of forces and to obtain comparable results with esthesiometers designed for other organs [28, 40]. We used force units to describe the sensory thresholds in our human tests.

### Endoscopic laser range-finder

To reduce observer variability when positioning the endoscope in the laryngopharyngeal tract and estimating the stimulus distance and impact site, which is required to accurately elicit laryngo-pharyngeal reflexes, we designed an endoscopic laser range-finder with the following specifications:

1. Precise and accurate distance measurements in a range from 2 to 12 mm. The error of measurement should be lower than the change of distance producing a >10 % change in the superficial pressure of air-pulses.
2. Rapid calculation of distances (calculation time <1 s) and low computer resource consumption to avoid interferences with image processing.
3. Wavelengths between 500 to 700 nm, visible to the human eye (to estimate the point of impact of the air-pulses over the mucosa), and adjustable power in the range of class 3R laser products of the IEC 60825 standard (IIIa of the FDA 21CFR1040.10, maximum power at the exit of the optical fiber <5 mW), which is considered safe for skin and also for eyes if handled carefully with restricted beam viewing. This range of power is used in commercial laser pointers. The adjustable power serves to regulate the laser power according to the luminosity of the white endoscopic light so that the laser spot may be visible during the test.
4. Ability to plot a polar grid, including circles and a cross, to provide additional assistance in standardizing the distance, site and angle of stimulus impact and adjust for changes caused by laryngeal movements during the test.
5. Laser that can be conducted through an optical fiber attached to the distal end of the endoscope. The outer diameter of the optical fiber is 0.9 mm, and the attachment must be ultrathin to avoid discomfort to the patient when the endoscope is introduced through the nose.

**Fig. 4:**
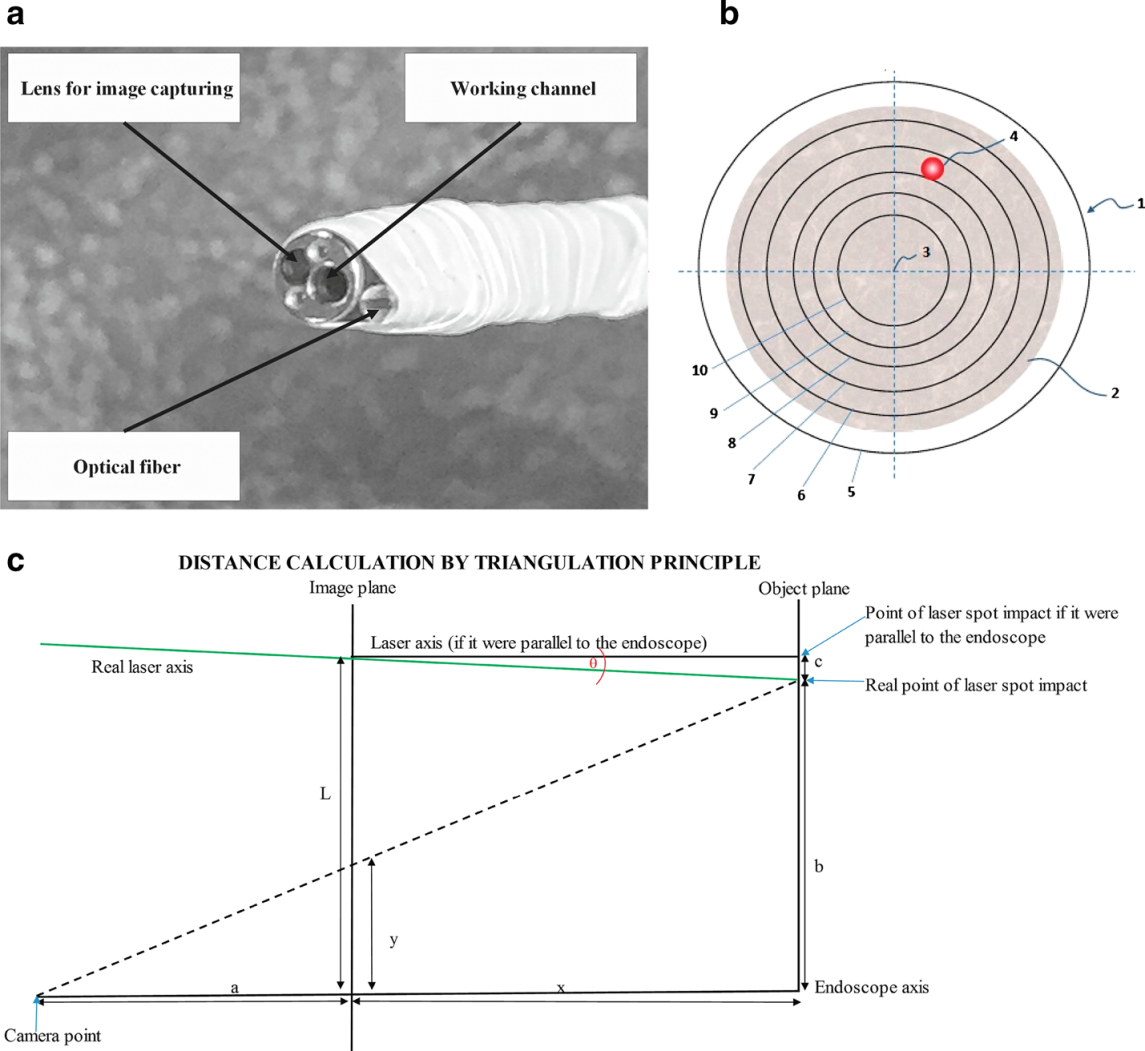
Optical fiber assembly, range-finder polar grid and distance calculation. **a** Laser optical fiber assembly at the endoscope distal end; **b** polar grid; and **c** distance calculation using a pinhole camera model [41]. *1* polar grid, *2* image captured from the target surface, *3* center of the polar grid (coincides with the center of the captured image); the polar grid includes circles corresponding to the estimated distances between the endoscope distal end and the target surface as follows: 5: 1.78 mm (used to center the endoscopic camera), *6* 3, 7:6, 8:9, 9:12, and 10:15 mm. *4* Laser spot. Distance calculation: *a* the distance used in the pin-hole camera model from the focal point (*camera point*) to the *image plane*; *b* the distance between the *real point of the laser spot impact* and the point of intersection of the camera model axis (*endoscope axis*) and the *object plane axis*; *c* the distance between the *point of impact of the laser spot if the laser axis were parallel to the endoscope* axis and the *real point of impact of the laser spot* (only the radial component is considered); *L* the distance between the laser optical fiber (*laser axis*) and the axis of the camera (*endoscope axis*) on the image plane; *x* the distance between the camera *image plane* and the *object plane* in millimeters; and *y* the distance on the *image plane* between the centroid of the laser spot and the center of the image in pixels (radius)

The esthesiometer includes a fiber-coupled diode laser module with a wavelength of 532 nm and output power configured to produce a laser beam power range from 1 to 5 mW. The image, including the laryngopharynx and the laser spot, is captured from the endoscope objective using a standard endoscopic camera; this image is used to permit endoscopic visualization of the laryngopharyngeal tract and to capture the laser spot needed to measure the distance between the endoscope tip and the site of stimulus impact. The optical fiber transmitting the laser was attached to the distal end of the endoscope and aligned with the center of the working channel and the distal end of the endoscope to maintain the same distance between the laser optical fiber core and the center of the lens for image capture through the endoscope (Figs. 2, 4).

Nevertheless, a slight misalignment can appear, as a perfect parallelism between both axes is very difficult to achieve, and should be considered by the system. The misalignment can come from two sources, that may appear alone or combined: (a) the laser optical fiber and the endoscope fiber lie on the same plane, but converge, or (b) they propagate on different parallel planes. In both cases, using cylindrical coordinates, the distance marked as b on Fig. 4c differs from the expected value, and must be corrected. Both effects are included on the sketch presented on this figure if we neglect the different azimuthal impact position for case (b), as it has no effect on the distance calculation.

*Distance measuring block* Once the laser is turned on, the laser spot on the surface in front of the endoscope distal end is recorded in a sequence of images. The position of the laser spot is then determined by using an image processing strategy. First, the sequence of images is mapped to a YUV luminance color space that allows a more salient representation of the laser spot. Then, a temporal background strategy is implemented to properly segment the spot and filter out the abrupt changes of illumination, which typically occur during the standard endoscopy. A temporal background is subsequently defined as the recursive average of the sequence of images, i.e., the pixels with less changes during the sequence. Each image is then compared with respect to the average image, and if the pixel exceeds τ times the average model, this pixel is considered to be noise and filtered out of the sequence. Ultimately, only the pixels with relatively constant illumination along the sequence are labelled as the laser spot. Similarly, several morphological operations, such as opening and closing, are conducted to define the region corresponding to the laser spot shape. The defined shape was then used to calculate the spot’s centroid. Subsequently, a Euclidean distance between the spot’s centroid and the endoscope distal end is defined as an index of the depth of the device based on the triangulation principle (Fig. 4). This triangulation principle was used to create an equation to calculate the distance between the endoscope distal end and the object where the laser spot impacts, and it includes the parameters depicted in Fig. 4 and defined below.

*a*: the distance used in the pin-hole camera model from the focal point (*camera point*) to the *image plane*.

*b*: the distance between the *real point of the laser spot impact* and the point of intersection of the camera model axis (*endoscope axis*) and the *object plane axis*.

*c*: the distance between the *point of impact of the laser spot if the laser axis were parallel to the endoscope* axis and the *real point of impact of the laser spot* (only the radial component is considered).

*θ*: tangent between the *endoscope axis* and the laser optical fiber axis (*laser axis*).

*L*: the distance between the laser optical fiber (*laser axis*) and the axis of the camera (*endoscope axis*) on the image plane.

*x*: the distance between the camera *image plane* and the *object plane* in millimeters (distance between the endoscope distal end and the object).

*y*: the distance on the image plane between the centroid of the laser spot and the center of the image in pixels (radius).

1 $$y/a= b /( a + x ) \to y = a \times b / ( a + x )$$

However,

2 $$\theta = c/x \to c = \theta \times x$$

3 $$b = L - c = L - \theta \times x$$

and

4 $$y = a \times (L - \theta \times x)/(a + x)$$

5 $$x = (a \times L - y \times a)/(y + a \times \theta )$$

where a, θ and L are geometrical parameters.

In Eq. (5), using L = 255.47, θ = 8.6675 and a = 8.353 produced R^2^ = 0.996 with a residual mean square = 0.15 and residual standard deviation (SD) = 0.13.

To improve the laser range-finder performance in terms of precision, accuracy and lower computer resource consumption, we set a learning process with several measurements comparing the distance between the endoscope distal end and the object where the laser spot impacts as explained in the distance measuring block below. Then, several regression functions were adjusted, and the best one was chosen.

**Fig. 5:**
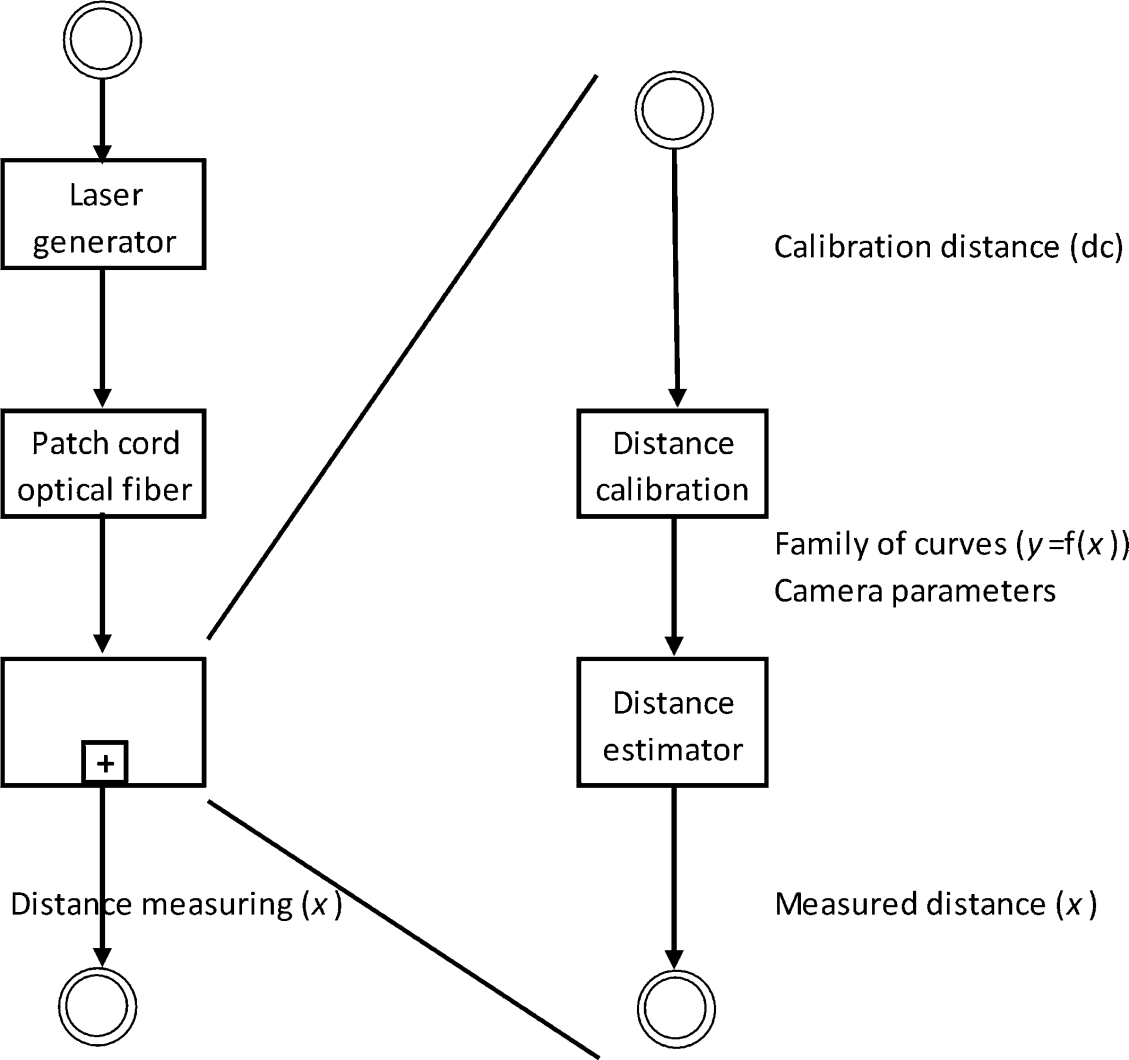
Distance measuring block

The distance measuring block (Fig. 5) has two modules that were developed by custom design software:

1. Distance calibration
2. Distance estimator

### Distance calibration

Input: calibration distance (*dc*)

Output: family of curves (*y* = f(*x*))

In this module, the objective was to set a learning process in which the calibration distance in mm (*dc*) between the endoscope distal end and the surface where the laser spot was projected was matched to the distance between the spot’s center of mass (spot’s centroid) and the center of the camera image in pixels, which is called the radius (*y*). The *dc* was measured using a high precision micrometer (Ball Stage 45MM-X-CNTR-SOL; Edmund Optics Inc.).

From these learned points, a set of regression functions were adjusted, including a linear regression, exponential regression, polynomial regression and other types of regressions. The goodness of fit of the regression equations was evaluated based on the coefficient of determination (R^2^) and residual analyses. The best regression equation in terms of its goodness of fit and lower computer resource consumption was the exponential regression (R^2^ = 0.994; residual mean square = 0.19; and residual SD = 0.14):

6 $$y = A *e^{{\left( {B *dc} \right)}}$$

In this equation, *y* is expressed in pixels and *dc* is expressed in mm. The constants *A* and *B* were obtained by regressing the experimental boundary conditions:

7 $$y = 232.73 *e^{ - 0.111 *dc} ;R^{2} = 0.994$$

### Distance estimator

Input: family of curves and camera parameters

Output: distance between the endoscope distal end and the surface of the mucosa where the laser spot is projected (*x*).

In this module, two processes are running in parallel: the spot processing and a polar grid plot with circles. The grid is drawn once.

Spot processing is developed as follows:

Spot segmentation

Spot center of mass computation

Radius normalization.

The distance is estimated by solving for the variable *x* from *y* in the curve (*y* = *f*(*x*)); thus, the reverse relationship between *y* (in pixels) and *dc* (in mm), as determined in the calibration, was used to derive *x* (in mm) by the following equation:

8 $$x = \frac{{\left( {\ln \left( {\frac{y}{A}} \right)} \right)}}{B}$$

We used the above exponential equation to plot a polar grid with circles as described in our protocol, which is published elsewhere [42]. As shown in Fig. 4b [42], polar grid 1 can be superimposed onto the real-time image 2 captured for the target tissue surface (center 3 of polar grid 1 coincides with the center of the image captured by the endoscope). The circles on the polar grid are used to indicate to the observer the estimated distances between the endoscope distal end and the target surface based on where the centroid of laser spot 4 visually falls within the polar grid while performing a visual inspection of the tissue surface. As shown in Fig. 4 [42], circles 5, 6, 7, 8, 9, and 10 represent estimated distances of 1.78, 3, 6, 9, 12, and 15 mm, respectively. The outermost circle indicates the shortest distance that can be measured by the laser range-finder and is used along with the center of the visual field to center the image captured by the endoscopic camera before starting an actual test. An additional cross was also plotted to cover the entire visual field and centered at the center of the visual field to assist in positioning the endoscope in the correct location of the laryngo-pharyngeal tract.

### Variables selected to evaluate the device’s reliability and verification

We performed the verification and evaluated the precision and accuracy of the device based on the pressure of the air-pulses, which is the variable triggering the mechanoreceptors [35]. Because the duration of air-pulses may potentially affect the pressure curve configuration of the air-pulses and the activation of mechanoreceptors, we also evaluated this characteristic [34]. Both variables were measured at the tube exit.

Based on the errors reported in previous evaluations of devices used to explore LPMS (approximately 20 % [34]), we aimed to improve the precision and accuracy to an error rate of <10 % in the LPEER. The verification aimed to demonstrate the following:

1. Air-pulse pressures measured within each category show low variability as indicated by a coefficient of variation (*CV,* defined as the ratio of the standard deviation to the mean) lower than 0.10;
2. Mean pulse pressure measured for each air-pulse category has a difference with the desired pressure lower than 10 %;
3. Air-pulse duration shows low variability as indicated by a *CV* lower than 0.10;
4. Mean air-pulse duration differs from the desired duration by less than 10 %.

### Human tests

We performed preliminary observations of the device performance using 10 subjects, which constituted a pilot validation of the test, to determine if there were additional factors affecting the reliability that were not controlled by the LPEER. This part of the study was conducted in accordance with Good Clinical Practice Guidelines and approved by the Institutional Review Board of the Universidad de La Sabana, and all of the subjects provided written informed consent.

These subjects received a standard clinical evaluation by a pulmonary doctor and a speech language pathologist who both had more than 7 years of experience in swallowing disorders. The patients underwent a standard fiberoptic endoscopic evaluation of swallowing with sensory testing (FEESST) [16], the endoscope was lubricated with hydrosoluble gel to decrease discomfort, and anesthetics were not used [16]. The sensory test was performed by an expert (more than 7 years of experience in FEESST) and a novice observer (1 month of training in FEESST) in a blinded manner. The severity of swallowing alterations was rated according to the findings of the food challenge on the FEESST and the dysphagia severity scale [16, 43]. Details of the protocol, including the standardization of the stimulus and distance of delivery, are published elsewhere [42]. In this pilot validation, examiners were chosen at the extreme of expertise (one expert and one novice) to increase the probability of detecting expert subjectivity determinants, which could limit the application of the test by examiners with lower degrees of expertise. Furthermore, observations by a novice examiner provide the additional advantage of better anticipating the performance of examiners introducing a new test in their clinical practice setting.

### Statistical methods

The pressure and duration of the air-pulses were the dependent variables analyzed as quantitative continuous variables, and statistic tests were performed to evaluate whether these variables were normally distributed.

To determine the factors affecting the superficial pressure, bivariate regression analyses were performed between each potential factor, which were the independent variables and the outlet pressure and superficial pressure, which were the dependent variables. Any independent variable associated with the dependent variable with a value of P < 0.25 was introduced in a multiple linear regression model to identify the variables that were independently associated with the dependent variable. Collinearity, interaction and residual analyses of the model were performed.

We selected the coefficient of variation (*CV*) as the statistic to determine the precision (repeatability) of the pressure and duration of the air-pulses because a correlation was observed between the SDs and the means for these variables [44, 45]. To assess the accuracy, the measured pressure and duration of the air-pulses were compared with the desired values.

The statistical analysis was performed using IBM-SPSS statistics software, version 20 (Armonk, NY, USA); R, version 2.14 (R Foundation, Wirtschaftsuniversität Wien, Vienna, Austria) and Microsoft Excel 2007 (Microsoft Corporation, Redmond, WA, USA).

## Results

### Factors determining the variability of air-pulse pressure

Several factors related to pressure regulator specifications have not been provided control mechanisms in previous devices, including the supply pressure (inlet pressure) range and hysteresis [46, 47]. In fact, these devices are not able to detect a supply pressure that is out of range [30, 34] and out of control hysteresis variability because air-pulses may be delivered at increasing, decreasing or random orders of pressure [30, 34, 46].

In the bivariate and multivariate analyses, we found additional factors that had a significant impact on the outlet pressure, including the voltage supplied to the pressure regulator (P < 0.001), the diameter of the tube (P < 0.001) and the duration of the air-pulses (P < 0.001), and the relative importance of these factors as measured by the standardized regression coefficients (beta coefficients) were 0.92, 0.12 and 0.30, respectively. The principal determinants of the superficial pressure were the outlet pressure (P < 0.001), the distance (P = 0.04) and the angle (P < 0.001) between the end of the tube and the surface, with beta coefficients of 0.96, −0.07, and −0.60, respectively.

When the endoscope distal end was placed at a distance of 2 mm from the site of impact, a change in the angle from 0 to 30 degrees decreased the superficial pressure by 39 % and a change from 0 to 60 degrees decreased the superficial pressure by 78 %. This effect was even greater at longer distances.

A change in distance of 3 mm or less decreased the superficial pressure by less than 5 %. However, changes in the distance between the endoscope and the sensor from 4 to 10 mm decreased the superficial pressure from 7 to 18 %.

**Fig. 6:**
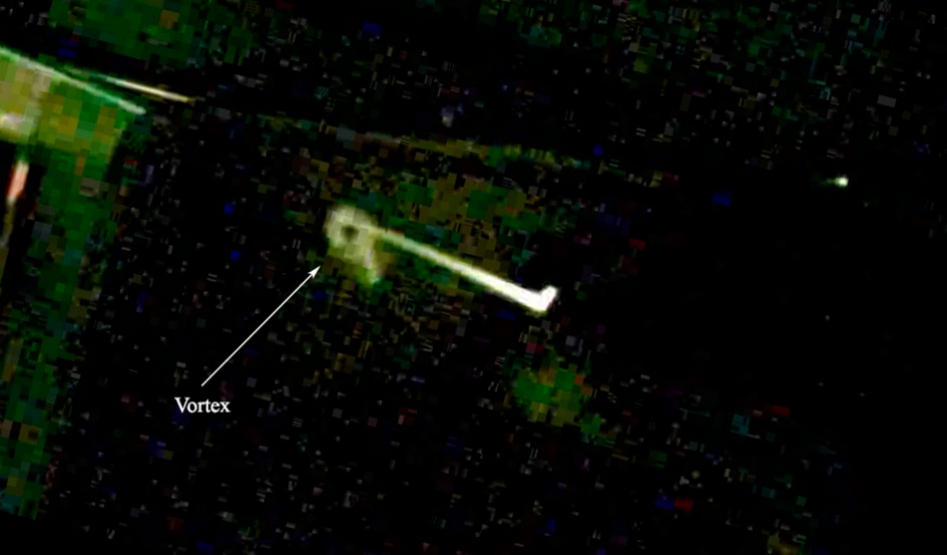
Air-pulse visualization in a dark room using chemical fog. The air-pulse morphology is similar to a *vortex ring*

The mean core diameter of the air-pulses was 4.6 mm, and its mean cortex diameter was 9.6 mm, both of which are greater than the hole of the sensor used for calibration of previous devices (1 mm). This increase in the diameter of the air-pulse when leaving the tube and its appearance when visualized using chemical smoke were similar to a vortex ring (Fig. 6).

### Verification and reliability of the air-pulse pressures

We found that the global *CV* for air-pulse pressures was 0.02 and for air-pulse durations was 0.06 (Table 1). The median pressure error was 2 % (comparing desired vs. measured pressures) and the median duration error (comparing desired vs measured durations) was −2.6 % (Table 1).

**Table 1:** Precision and accuracy of the air-pulse pressure and duration at the tube outlet

	Air-pulse pressure	Air-pulse duration
CV	0.02	0.06
Median difference between desired and measured value (%)	−0.3	−2.6
IQR (%)	−2.0 to 0.8	−4 to 0.1

*CV* coefficient of variation, *IQR* interquartile range

To overcome the limitations of previous sensors used to measure the stimulus triggering laryngo-pharyngeal reflexes caused by the air-pulse diameter [29, 34], which was more than four-times greater the diameter of the sensor hole, we used an analytical balance with a 100 mm pan diameter (Precisa BJ 100 M, Precisa Gravimetrics AG, Dietikon, Switzerland) to characterize the air-pulses in terms of mass and force and to obtain comparable results with esthesiometers designed for other organs [28, 40]. We used force units to describe the sensory thresholds in our human tests.

### Verification and reliability of the endoscopic laser range-finder

**Fig. 7:**
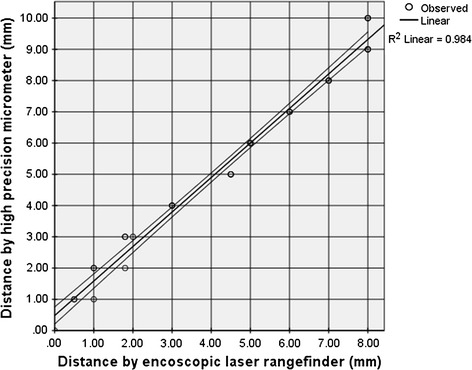
Distance determined using the endoscopic laser range-finder (telemeter) vs distance determined using the high-precision micrometer. The *middle line* represents the linear regression line and the *upper* and *lower lines* represents its 95 % confidence interval (95 % CI)

The average difference between the distance measured by the laser range-finder and the distance measured by the high precision micrometer was 0.9 ± 0.4 mm. The best performance of the laser range-finder was in the range of 4–9 mm, with differences below 1 mm between the distance measured by the laser range-finder and the micrometer (measurement error <10 %). Considering the aforementioned effect of distance on the air-pulse pressure, an error of ±1 mm on the distance estimation would change the superficial air-pulse pressure by ±2 %. A comparison of the measurements made using the laser range-finder and the high precision micrometer in a range from 1 to 10 mm showed a linear relationship (Fig. 7) according to the following equation:

9 $$d_{m} = 1.105 \times d_{\text{lr}} + 0.48$$

where *d*_*m*_ corresponds to the distance measured using the high-precision micrometer in mm and *d*_*lr*_ corresponds to the distance measured using the laser range-finder in mm.

The Pearson correlation coefficient between the distance measured using the laser range-finder and the distance measured using the micrometer was 0.99 (P < 0.001), and the coefficient of determination (R^2^) was 0.98.

### Evaluation of human subjects

We evaluated 10 subjects, including patients with dysphagia secondary to ischemic stroke, gastroesophageal reflux disease and irritable larynx and normal controls (Table 2).

**Table 2:** Characteristics of patients evaluated by FEESST

Subject	Sex	Age	Diagnoses	Dysphagia severity	Right LART (mN)	Left LART (mN)	Right CRT (mN)	Left CRT (mN)	Right GRT (mN)	Left GRT (mN)
1	M	49	Healthy control	Normal	0.2	0.2	4.0	3.4	4.0	3.4
2	F	50	Healthy control	Normal	0.1	0.04	4.0	4.0	2.4	2.4
3	F	47	GER, no dysphagia symptoms	Normal	0.3	0.3	4.0	4.0	1.0	Absent reflex
4	M	40	GER; dysphagia symptoms, epilepsy	Normal oropharyngeal swallowing	0.3	0.3	4.6	2.4	Absent reflex	Absent reflex
5	M	84	Ischemic Stroke	Mild	0.5	0.5	Absent reflex	Absent reflex	Absent reflex	Absent reflex
6	M	64	Ischemic Stroke	Mild	0.4	0.5	Absent reflex	Absent reflex	Absent reflex	Absent reflex
7	F	68	Ischemic Stroke	Moderate to severe^a^	Absent reflex	Absent reflex	Absent reflex	Absent reflex	Absent reflex	Absent reflex
8	M	84	Ischemic Stroke	Moderate to severe^a^	Absent reflex	Absent reflex	Absent reflex	Absent reflex	Absent reflex	Absent reflex
9	M	34	Irritable Larynx	Normal	0.3	0.3	0.3	0.3	1.4	1.4
10	F	57	Irritable Larynx	Normal	0.3	0.3	1.0	1.0	0.3	1.9

*FEESST* Fiber-Optic Endoscopic Evaluation of Swallowing With Sensory Testing, *LART* laryngeal adductor reflex threshold, *CRT* cough reflex threshold, *GRF* gag reflex threshold, *F* female, *M* male, *GER* gastroesophageal reflux, *Absent reflex* the reflex was not triggered by the air-pulse of maximum intensity

^a^Aspiration of 5–10 % of bolus to the lungs

Two observers evaluated these subjects: one expert with 9 years of experience in FEESST and one novice with one month of training in the test. Each observer performed two measurements per side of the laryngo-pharyngeal tract of the LART (laryngeal adductor reflex threshold).

The expert observer measured the CRT (cough reflex threshold) and GRT (gag reflex threshold) of the laryngo-pharyngeal tract twice per side. The CRT was measured at the same site of the LART, although compared with the LART, the CRT required air-pulses of greater intensity and duration [42]. The GRT was measured by delivering air-pulses of the same intensity and duration as those used for the CRT but delivered just lateral to the epiglottis. Details of the protocol are published elsewhere [42].

A total of 80 measurements were performed for the LART, and the subjects underwent 80 additional measurements corresponding to the CRT and GRT. The intra- and inter-observer differences in the LART results were used to determine whether there were additional factors affecting the test reliability that were not yet controlled and showed a median difference in the intra- and inter-observer LART measurements of 0.0 mN and an Interquartile Range (IQR) of 0.0–0.20 mN (Table 3).

**Table 3:** Intra-observer and inter-observer absolute differences in the LART results

Number of measurements	80 (mN)
Intra-observer absolute difference: median (IQR)	0.0 (0.0–0.1)
Inter-observer absolute difference: median (IQR)	0.0 (0.0–0.2)

*IQR* interquartile range; the differences in the measurements are expressed as absolute differences: ($\sqrt {difference^{2} } )$

We observed a median LART of 0.14 mN in normal subjects (corresponding to 2.5 mmHg in the MPX sensor) and a dose–response gradient with a higher LART in those patients with more severe dysphagia. Patients with aspiration secondary to stroke had severely compromised laryngo-pharyngeal sensitivity with an absence of LART, CRT and GRT. Patients with irritable larynx had mildly increased LART but lower CRT and GRT (Table 2).

The subjects reported mild to moderate discomfort with no pain during the test, and there were no other adverse events.

## Discussion

Our approach of first studying the factors affecting the reliability of the stimuli triggering laryngo-pharyngeal mechanoreceptors proved to be useful in finding additional factors to those previously described [34] that have an influence on the reliability of air-pulses used to measure the LART. Controlling such factors may improve the reliability of LPMS measurements. The principal determinant of the superficial pressure, which is responsible for stimulating mechanoreceptors, was the outlet pressure of the air-pulses. This outlet pressure was determined by the voltage supplied to the pressure regulator, the diameter of the tube conducting the air-pulses and the duration of the air-pulses. Insufficient control of factors related to pneumatic valve specifications, such as the supply pressure and hysteresis, may also induce a loss of reliability on air-pulse pressure. In addition, we found that it is not sufficient to control the variability of outlet pressure because the superficial pressure of the air-pulses is also significantly affected by factors such as the distance and angle of impact, which have not been controlled by current laryngo-pharyngeal esthesiometers using systems independent of observer subjectivity. The addition of an endoscopic rangefinder and optical grid to reduce observer subjectivity when estimating the distance and angle of impact to the control mechanisms of the outlet pressure allowed us to obtain reliable air-pulses and reliable sensory thresholds in the pilot validation.

To our knowledge, this is the first study that has measured the relative importance of the conducting tube diameter, air-pulse duration, and distance and angle of impact, and these factors are all required to obtain reliable stimuli for LPMS tests. By controlling all of the aforementioned factors, as well as the supply pressure and hysteresis, we were able to improve upon the reported stimulus reliability of previous devices [34] and surpass our goals. Our LPEER showed good precision (*CV* = 0.02) and accuracy, with differences of 0.3 % between the desired and measured pressures and 2.6 % between the desired and measured duration, and these results indicate that precise and accurate air-pulses could be administered for the exploration of mechanosensitivity. Our device incorporates an endoscopic laser range-finder and a visual field grid, components that have not been included until now in endoscopic esthesiometers. Excellent correlations were observed between the distances calculated by the endoscopic range-finder and distances measured using a high-precision micrometer as indicated by the regression equation and its R^2^ value. This characteristic allows the LPEER to resolve issues with observer subjectivity when estimating the distance, site and angle of impact of the air-pulses. All of these factors are important determinants of the superficial pressure at the site of impact of the air-pulses over the mucosa, although they had not been controlled by previous devices used for LPMS tests [30, 34].

Because of the diameter of the air-pulses determined in this study, calibration using a sensor with a hole smaller than the endoscope diameter would be unreliable, and a more feasible solution is to use a sensor system that is connected to the exit of the endoscope or tube conducting the air-pulses and has a diameter that is at least equal to the external diameter of the endoscope or tube. Furthermore, the “Y” open system, which has been used for the calibration of previous devices, does not measure the pressure at the exit of the tube or endoscope conducting the air-pulses but rater at the exit of a tube of similar length and diameter connected by a “Y” connector [31, 34]. If the specifications of the tube conducting the air-pulses change (e.g., diameter, length, material, or notches because of bronchoscope damage, thus causing partial obstruction of the conducting tube), the pressure measured at the sensor system would not correspond to the outlet pressure. Constant specifications must be maintained for the air-pulse generation and conducting components of the LPEER. Additionally, periodic measurements of the air-pulse pressure at the exit of the conducting tube should be performed to ensure appropriate device calibration. Such measurements are more reliable than simultaneous measurements performed during the test with a sensor system that is unable to detect the effect of unnoticed changes in the conducting system on the air-pulse pressures.

We developed a protocol for measuring the LART using the LPEER that includes the precise localization of the delivered stimulus with the aid of an endoscopic laser range-finder and visual grid as well as a standardized sequence of stimulus administration. Our pilot validation included observers with considerable differences in expertise to include extremes of experience in the test, which will allow us to better anticipate potential problems when introducing this test in novel scenarios. In this validation, which was performed on subjects affected by various conditions, we found similar results in the intra- and inter observer repeated measurements, suggesting that other clinical factors with potential effects on the reliability of the test might be of lower importance than the factors directly or indirectly controlled by the LPEER and indicating that the performance of the test by observers with low levels of experience is likely feasible. However, the sample used in this pilot validation was small and not appropriate for including all of the validation tests; therefore, this finding must be confirmed in a clinical validation study with a larger sample of subjects.

In our human tests, we found sensory thresholds (0.14 mN or 2.5 mmHg) similar to those reported by Avid in healthy subjects [31, 48]. Our results are also comparable with those reported by Grushka, who found a mechanoreceptor sensory threshold of 14.9 mg (equivalent to 0.15 mN) for the most sensitive part of the tongue [40].

We also found a dose–response gradient with greater sensory compromise in patients with more severe dysphagia. These results are consistent with previous studies [10] and highlight the potential utility of the LPEER for the development and perfection of novel therapies aimed at improving dysphagia associated with sensory deficits [49]. Our novel test for measuring the CRT and GRT could detect higher thresholds for these reflexes in stroke patients with dysphagia. This finding is consistent with previous evidence showing a compromise of protecting cough in stroke patients [50, 51]. However, we have not found previously designed tests for evaluating the CRT or GRT via stimulation of the laryngo-pharyngeal mechanoreceptors through standardized air-pulse stimuli. Our work is an attempt to fill this void and help identify more effective treatments against dysphagia and its complications, such as pneumonia, malnutrition and death. Furthermore, the more comprehensive evaluation of sensory compromise provided by the LPEER might be useful for characterizing the sensory impairment of OSA [4, 5] and finding alternatives to CPAP in the treatment of this condition.

Our patients with irritable larynx had a lower CRT and GRT despite having mildly increased LART. This finding, if confirmed in a prospective diagnostic accuracy study, could help differentiate irritable larynx from other causes of chronic cough and identify more effective treatments for this common cause of chronic cough.

The CRT, GRT, LART protocols were well tolerated by subjects and did not cause any relevant adverse events. Patients did not have any discomfort caused by noise or general functioning of the LPEER. This finding is consistent with previous reports on FEESST safety [52], although the safety of introducing a higher stimulus for the CRT and GRT would require confirmation in a prospective clinical study.

## Conclusions

We designed, developed and performed a pilot validation of a novel device capable of delivering precise and accurate stimuli for the exploration of LPMS and generated standard protocols for measuring LART, CRT and GRT. Upon successful testing in a clinical validation study, these developments could aid in the study, diagnosis and treatment of a great number of patients suffering from disorders affecting laryngo-pharyngeal sensitivity, such as dysphagia, OSA, irritable larynx, voice disorders and chronic cough.

## Abbreviations

95 % CI: 95 % confidence interval
CRT: cough reflex threshold
CV: coefficient of variation
*dc*: calibration distance
FEESST: fiberoptic endoscopic evaluation of swallowing with sensory testing
GER: gastroesophageal reflux
GRT: gag reflex threshold
IQR: interquartile range
LART: laryngeal adductor reflex threshold
LPEER: laryngo-pharyngeal endoscopic esthesiometer and range-finder
LPMS: laryngo-pharyngeal mechano-sensitivity
OSA: obstructive sleep apnea
PAF: population attributable fraction
SD: standard deviation
YLLs: years of life lost
